# Virtual Screening and Meta-Analysis Approach Identifies Factors for Inversion Stimulation (Fis) and Other Genes Responsible for Biofilm Production in *Pseudomonas aeruginosa*: A Corneal Pathogen

**DOI:** 10.3390/cimb46110770

**Published:** 2024-11-13

**Authors:** Promise M. Emeka, Lorina I. Badger-Emeka, Krishnaraj Thirugnanasambantham

**Affiliations:** 1 Department of Pharmaceutical Science, College of Clinical Pharmacy, King Faisal University, Al Ahsa 31982, Saudi Arabia; 2 Department of Biomedical Science, College of Medicine King Faisal University, Al Ahsa 31982, Saudi Arabia; lbadgeremeka@kfu.edu.sa; 3 Pondicherry Centre for Biological Science and Educational Trust, Sundararaja Nagar, Puducherry 605004, India

**Keywords:** bacterial keratitis, biofilm, fis, genes, meta-transcriptome analysis, *Pseudomonas aeruginosa*

## Abstract

Bacterial keratitis caused by *Pseudomonas aeruginosa* is indeed a serious concern due to its potential to cause blindness and its resistance to antibiotics, partly attributed to biofilm formation and cytotoxicity to the cornea. The present study uses a meta-analysis of a transcriptomics dataset to identify important genes and pathways in biofilm formation of *P. aeruginosa* induced keratitis. By combining data from several studies, meta-analysis can enhance statistical power and robustness, enabling the identification of 83 differentially expressed candidate genes, including fis that could serve as therapeutic targets. The approach of combining meta-analysis with virtual screening and in vitro methods provides a comprehensive strategy for identifying potential target genes and pathways crucial for bacterial biofilm formation and development anti-biofilm medications against *P. aeruginosa* infections. The study identified 83 candidate genes that exhibited differential expression in the biofilm state, with fis proposed as an ideal target for therapy for *P. aeruginosa* biofilm formation. These techniques, meta-analysis, virtual screening, and invitro methods were used in combination to diagnostically identify these genes, which play a significant role in biofilms. This finding has highlighted a hallmark target list for *P. aeruginosa* anti-biofilm potential treatments.

## 1. Introduction

A dangerous and potentially blinding corneal infection, the transparent anterior layer of the eye that encompasses the iris and pupil, is called bacterial keratitis. Bacteria enter the cornea and cause infection, which results in inflammation and damage. The adaptable opportunistic pathogen *P. aeruginosa* may result in numerous problems, including corneal infections. There are numerous virulence factors in *P. aeruginosa* that aid in its establishment and infliction of harm in the host tissue when it comes to corneal infections. The production of biofilms and cytotoxicity to cornea have been directly connected to *P. aeruginosa*’s enhanced resistance to antibiotics [1].

*P. aeruginosa* strain PAO1 is a well-known model organism for studying biofilm development and related activities. Because PAO1 is well-known for its capacity to build durable biofilms, it is an invaluable tool for comprehending the physiological and molecular components of biofilm formation [2]. Attachment and early colonization are two important characteristics of PAO1 biofilms [3]. To begin the process of forming a biofilm, PAO1 uses pili and specific adhesins to adhere to surfaces. Such factors as flagella and type IV pili mediate initial adhesion [4]. Extracellular Polymeric Substances (EPS) components, such as pellicle polysaccharide (Pel), alginate, and polysaccharide synthesis locus (Psl), are produced by PAO1 and are essential for the stability and structure of biofilms. Antimicrobial drugs and environmental pressures are protected from by EPS [5]. PAO1 controls gene epression linked to biofilm development and coordinates the formation of biofilms by means of Quorum Sensing (QS) systems, namely the Las, PQS, and Rhl systems. In addition, QS regulates the synthesis of EPS components and virulence factors [6]. PAO1 biofilms have a complex architecture that includes microcolonies, water channels, and blank spaces [7]. PAO1 biofilm formation is controlled by sophisticated regulatory networks involving various transcriptional regulators (e.g., FleQ and AlgU), cyclic-di-GMP signaling, and two-component systems (e.g., GacS/GacA) [8].

Biofilm formation and gene expression are influenced by variables such as nutrients supply, oxygen levels, and surface characteristics. During biofilm formation, metabolic changes take place that result in changed gene expression and the creation of EPS [9]. The rate of metabolism of PAO1 biofilms is less than that of planktonic cells. Pathogenic biofilms vary depending on the species and strain; they differ from planktonic cells among a given species in a number of ways. The persister cell (PC) is a temporary reversible phenotype that is inactive and resistant to drugs. Cephalosporin therapy alone caused PC formation in the planktonic stage of P. aeruginosa, whereas cephalosporin, aminoglycoside, and fluoroquinolone caused PC formation in the biofilm stage [10]. Because of resistant cells and decreased antimicrobial agent penetration, PAO1 biofilms in comparison to planktonic cells exhibit enhanced tolerance and resistance to antibiotics [11]. In clinical settings, biofilm-associated antibiotic resistance poses a serious problem. PAO1 biofilms have been linked to persistent infections linked to wounds, medical device-related illnesses, and cystic fibrosis [10,12]. The discovery of innovative treatments targeting biofilm-specific mechanisms such as EPS production and QS is aided by an understanding of PAO1 biofilm biology. In order to increase the effectiveness of antibiotics, research is concentrated on breaking down biofilms using antimicrobial peptides and nanoparticles [13,14,15]. *P. aeruginosa* PAO1 biofilms have a complex architecture, produce EPS, and use quorum sensing and metabolic adjustments to regulate themselves. Studying PAO1 biofilms provides vital insights into biofilm biology and antibiotic resistance mechanisms. This has implications for creating effective tactics to combat diseases linked to biofilms [16].

Distinctions between planktonic and biofilm bacteria are the main justification for the creation of anti-infective methods against biofilms [17]. Numerous approaches, from molecular ones like Northern blotting and PCR [18,19,20] to high-throughput ones like proteomics and transcriptomics, are used in research to find key biofilm genes [21,22,23]. However, because of the variations in the methods employed and the strain dependence of the molecular components, the collection of discovered genes found in every study varies and exhibits minimal overlap between one another [24]. Many biological issues, including the pathogenicity of bacteria like *P. aeruginosa*, can be studied by analyzing gene expression changes and examining the underlying mechanisms using datasets from transcriptomic technologies like RNA-seq and microarray [25]. Better, more trustworthy results are obtained from extensive expression profile analyses that include a greater quantity of samples, yet organizing such an experiment is not always possible. A meta-analysis approach is used in these situations, which includes the datasets and findings from numerous earlier investigations. This method removes inconsistencies, lifts the sample size limit, and finds genes that are consistently changed in different research, all while increasing statistical power [24].

A meta-analysis of transcriptome data related to *P. aeruginosa* biofilms entails collecting and analyzing various research to uncover similar trends and key genes/pathways involved in biofilm formation. Utilizing meta-analysis and feature selection, the current study aims to identify a signature collection of putative genes that distinguish significantly in *P. aeruginosa* biofilms utilizing transcriptome data obtained from various investigations. To discover differential expression, random-effects modeling was applied to public gene expression datasets of *P. aeruginosa* in planktonic and biofilm settings. Additional feature selection techniques were used to identify potential genes involved in biofilm development, which were confirmed using incredibly accurate classifiers. In addition, based on the data from meta-analysis, virtual screening and invitro approaches were applied to identify a *P. aeruginosa* biofilm inhibitor.

## 2. Materials and Methods

### 2.1. Chemicals, Microbial Strains and Culture Conditions

The *Pseudomonas aeruginosa* (MTCC1688) was acquired from the Institute of Microbial Technology in Chandigarh, India. It was cultivated on Nutrient Agar Medium (NA; cat. no. MM012; Himedia, Maharashtra, India) and kept at 4 °C. Brain Heart Infusion (BHI) broth was inoculated with a single colony of *P. aeruginosa*. (Cat No. M210 Himedia, Maharashtra, India), and then the suspensions were incubated for an entire night in a shaker at 200 rpm and 37 °C. Himedia, Maharashtra India supplied the dexamethasone (at No. C80687; 99% purity). All chemicals and media utilized in the current investigation, unless otherwise noted, were purchased from Himedia (Maharashtra, India) and Merck (Bengaluru, India).

### 2.2. Dataset Collection

Using keywords, the National Centre for Biotechnology Information’s public expression profile database Gene Expression Omnibus (GEO) was searched for expression records of *P. aeruginosa* planktonic and matching biofilm forms. Platform variations and sample preparations are mostly responsible for significant variations observed in comparable experiments that distinguish between two conditions. As a result, only samples from the same studies and platform that met both test and control conditions were retrieved. Four datasets, derived from two distinct methodologies (microarray and RNA sequencing), were recognized (Table 1).

### 2.3. Data Processing and DEG Screening

In order to perform the meta-analysis and identify shared DEGs within the expression profiles, Network Analyst, an online tool for integrative meta-analysis, was used [25]. The ComBat approach was used to remove the batch effects after the normalized expression profiles in the required format were uploaded. The random effect size in the meta-analysis was selected in accordance with the outcomes of the Cochran’s Q-test, which estimates statistical heterogeneity [26,27]. The DESeq2 tool in the R programming 1.47.0 was used to perform differential expression analysis of the datasets; metrics such as *p*-values, *p*-value adjusted, and Log2 Fold Change (Log2FC) were analyzed for each gene.

### 2.4. Gene Ontology and Pathway Analysis

The annotation, visualization, and integrated discovery (DAVID) online database was utilized to analyze the resulting meta-DEGs for gene ontology (GO), which covers biological process, cellular component, and molecular function as well as pathway enrichment [28,29]. DAVID analysis yielded functional annotation clustering and Kyoto encyclopedia of genes and genomes (KEGG) pathways. Biological process-related clusters were combined when the enrichment score (ES) threshold was set at 0.5. The enriched GO pathway was visualized using Hiplot (ORG) [30].

### 2.5. Virtual Screening for Identification of Drug Candidate

The target proteins’ (3-D) experimentally determined structures (fis; PDB id: 6m10) were obtained from the Protein Data Bank (https://www.rcsb.org/, accessed on 18 April 2024) [31]. UCSF Chimaera v1.16’s conjugate gradient and steepest descent algorithms were employed to minimize the energies of fis protein following the addition of polar hydrogen atoms and other missing atoms, the removal of heteroatoms and co-crystallized water, and the assignment of partial charge [32]. The produced receptor was uploaded to the DrugRep webserver to perform receptor-based virtual screening [33]. Pocket 1 was chosen to be docked (center coordinates: −17.0, −8.2, and 9.4, size values: 23, 19, and 16). The library chosen for virtual screening was the Approved Drug Library. The selected ligand (dexamethasone) was reanalyzed for its interaction with fis using the Autodock 4.2.6 tool. To elucidate the pattern of interactions between the fis and dexamethasone, Discovery Studio Visualizer 2022 was used 27. The fis in complex with dexamethasone was examined in terms of their binding affinity, conformation, and interactions (docking score). Using the CABS-Flex 2.0 system, which relies on coarse-grained protein movements, molecular dynamics (MD) simulations of complex and the native protein (fis) were carried out [34]. There were other distance constraints, such as a global weight of 1.0, in addition to more than 50 cycles and 50 trajectory frames in 10 ns each. The complexes’ mobility was represented in terms of root-mean-square fluctuations (RMSF).

### 2.6. Antibacterial Activity of Dexamethasone Against P. aeruginosa

Using a well-diffusion approach, the antimicrobial effect of dexamethasone against *P. aeruginosa* was analyzed [35,36]. Nutrient agar (Himedia, Maharashtra, India) was used to cultivate *P. aeruginosa* for a duration of 24 h at 37 °C. A 0.85% NaCl (*w/v*) sterile saline solution was used to provide inoculations of bacteria equivalent to 0.5 McFarland (1 × 10^8^ CFU/mL). The aforementioned suspension was distributed on Mueller–Hinton agar (MHA) plates. A 6 mm diameter sterile disc was placed on the agar medium and loaded with dexamethasone (0.5, 1, 2, and 4 mg/disc). As a positive control, a disc containing 20 µg ciprofloxacin was placed on the plates and incubated at 37 °C for 24 h. The zone of inhibition (mm in diameter) was used to measure the activity against *P. aeruginosa*.

### 2.7. Minimal Inhibitory Concentration (MIC)

Planktonic suspended cells were used to estimate the least inhibitory concentrations (MICs) of *P. aeruginosa* using broth micro-dilution on microtitre plates [37]. The plates were made using aseptic methods. After pipetting 100 µL of each chemical, dexamethasone (100 mg/mL), in DMSO into each well, the two substances were serially diluted to their lowest concentrations, 0.625 mg/mL and 1.25 µg/mL, respectively. Following that, 10 µL of bacterial suspension (1 × 10^6^ CFU/mL) was added to the wells, and the mixture was kept at 37 °C for 24 h. Once incubated, 30 µL of resazurin (0.015%) was added to each well, and the wells were incubated for a further two to four hours to observe any color changes. After measuring plate absorbance at 570 nm, the quantity of dexamethasone/ciprofloxacin that suppressed cell growth was named as the minimum inhibitory concentration (MIC).

### 2.8. Inhibition Assay in Biofilm Formation

The ¼ MIC of dexamethasone was treated with suspensions of bacteria in BHI broth enriched with 1% (*w/v*) sucrose (1 × 10^7^ CFU/mL) for 48 h at 37 °C in a 96-well cell culture plate with a flat bottom. The addition of sucrose aided in the development of bacterial biofilms [38]. In addition to the dexamethasone treatment, appropriate culture and media controls were included. Any residual cells that were not adherent to the biofilm were then eliminated by rinsing with sterile PBS after the medium and the supernatant carrying dispersed planktonic cells were removed. The potential of dexamethasone to suppress biomass and metabolism on *P. aeruginosa* biofilm was ascertained through the use of the XTT assay and crystal violet staining. For the XTT experiment, 100 µL of fresh, sterile medium was added to each well. Subsequently, 50 µL of a detection solution containing XTT (2,3-Bis-(2-Methoxy-4-Nitro-5-Sulfophenyl)-2H-Tetrazolium-5-Carboxanilide) and phenazine methosulfate (PMS) was introduced into each well, and it was incubated for four hours at 37 °C. The absorbance at 450 nm was then measured after the plates were rapidly agitated for 10 s to mix the dye with the solution [39]. In order to evaluate the dexamethasone’s capacity to inhibit *P. aeruginosa* biofilm biomass, 100 µL of 0.1% *w/v* crystal violet solution was introduced into each well. After that, the wells were allowed to incubate for ten minutes. Each well was washed using sterile distilled water after the unattached crystal violet solution was removed. Next, to measure the absorbance of color intensity at 595 nm, each well received 95% ethanol, and the plate was shaken gently for 30 min at room temperature [40]. The following formula was used to determine the percentage of biofilm inhibition throughout the triplicate experiment:

Biofilm inhibition (%) = 100 − ((OD_570 in sample treatment_ − OD_570 in untreated bacteria_)/(OD_570 in untreated sample_ − OD_570 in untreated bacteria_) × 100)

### 2.9. Fluorescent Microscopic Analysis of Biofilm

The fluorescence microscopic studies of dexamethasone impacts on *P. aeruginosa* biofilms were performed as previously described, with a few minor variations [41,42]. In short, *P. aeruginosa* biofilms were formed on 12 well culture plates for 72 h at 37 °C using varied dexamethasone concentrations (ranging from 0.25 to 4 mg/mL) in BHI medium enriched with 2% (*w/v*) glucose. The wells were delicately washed with antiseptic phosphate buffered saline (PBS) before staining with acridine orange for 30 min. A fluorescent microscope (Optika, SRL, Ponteranica, BG, Italy) was utilized to record five arbitrary images, with the intensity of light, the background, and contrast remaining constant during the experiment.

### 2.10. Statistical Analysis

Each experiment was run three times in triplicate. Graphpad Prism 10.2 software was used to estimate the intergroup differences using one-way ANOVA and T-test. The mean and standard error mean (SEM) of the data are displayed. A *p*-value was considered statistically significant if it was less than 0.05.

## 3. Results

### 3.1. Dataset Selection and Quality Control

**Figure 1:**
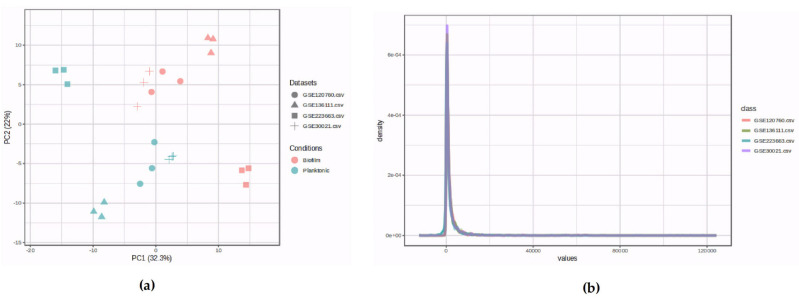
Data pre-processing and processing; (**a**) Comparison and contrast between the biofilm and planktonic samples using PCA plots of batch effect removal; (**b**) Density plots of batch effect elimination against log2 of read counts display the relative distribution of various counts within each group.

Initially, 4 expression profiling by high-throughput RNA sequencing and 2 expression profiling by microarray from biofilm and planktonic samples were retrieved from the GEO database. However, based on the initial principal component analysis (PCA), two high-throughput RNA sequencing datasets were removed from further analysis. It was determined that the four profiles with untreated planktonic and biofilm were appropriate for additional pre-analysis stages. The GEO accessions GSE30021, GSE120760, GSE136111, and GSE223663 corresponded to the chosen expression profiles. Table 1 contains comprehensive details about the chosen datasets. For the meta-analysis, 12 biofilm and 12 planktonic samples were chosen using PCA, outlier removal, normalization, and batch effect removal procedures (Figure 1a,b).

### 3.2. Meta-Analysis and DEG Identification

**Figure 2:**
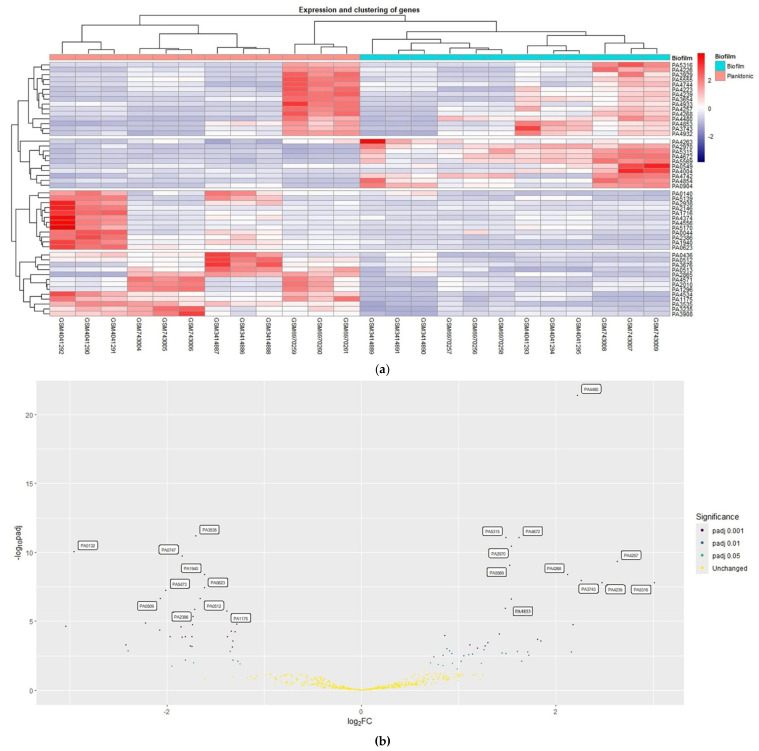
Analysis of GEOs. (**a**) The top 50 DEGs’ heatmap based on adjusted *p*-value; (**b**) Volcano plot of genes that were selected based on meta-analysis using random effect size.

It was successful to apply meta-analysis (random effect size) to the expression profiles’ pre-processed matrix files. Based on the findings of the Cochran’s Q-test and the literature review, the random effect size was chosen as the meta-analysis approach. Meta-analysis using random effect size revealed 530 genes were commonly expressed in all 4 expression profiles. Using an adjusted *p*-value of 0.05, 83 genes that include 45 upregulated and 38 downregulated genes were determined to be meta-DEGs (Table 2). Supplementary Table S1 has the entire list of the DEGs that have been identified. The heatmap of the top 50 meta-DEGs is shown in Figure 2a, and the volcano plot of DEGs is shown in Figure 2b. The top 50 meta-DEGs sorted by their effect sizes (fold changes) are listed in Table 3 and Table 4.

### 3.3. Functional Classification of DEGs Using Gene Ontology and KEGG Pathways Analysis

DAVID tools version 6.8 was used to perform functional annotation clustering in order to assess the genes that are expressed differently in *P. aeruginosa*’s biofilm and planktonic forms. Gene ontology (GO) analysis divides the three categories of differentially expressed genes into biological processes, molecular function, and cellular components.

**Figure 3:**
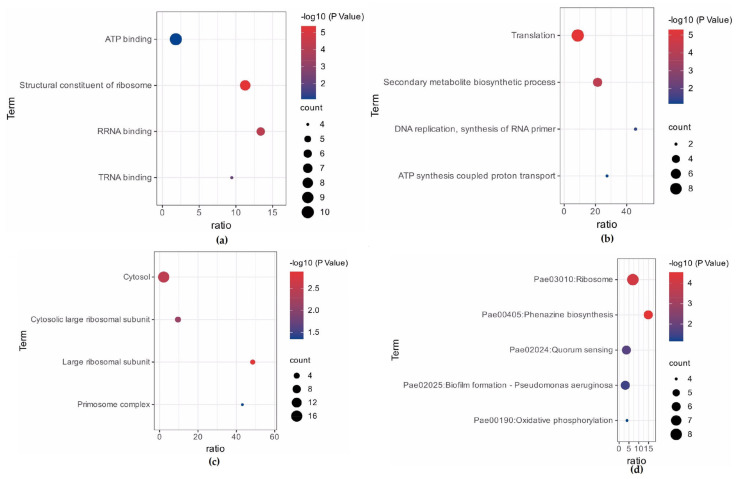
Functional enrichment analysis of DEGs up-regulated in biofilm samples. (**a**) Enriched gene GO terms under molecular function; (**b**) Enrichment GO terms under biological process; (**c**) Enriched GO terms under cellular component; (**d**) Enriched KEGG pathways.

**Figure 4:**
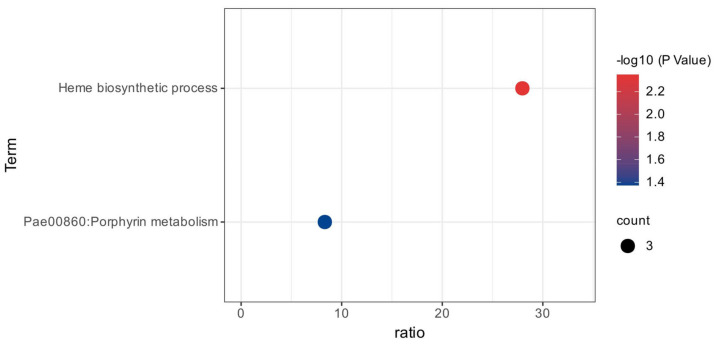
Functional enrichment analysis of DEGs down-regulated in biofilm samples.

Based on the enrichment score (ES), functional annotation clustering created a total of 4 clusters for up-regulated and 2 clusters for down-regulated genes from the 83 DEGs of Biofilm. In the case of up-regulated genes, the most enriched clusters consisted of genes associated with translation (ES = 3.89) and secondary metabolite biosynthetic processes (ES = 2.40). Further functional enrichment analysis of DEGs for up-regulated in biofilm samples revealed enrichment of molecular function (ATP binding, structural constituent of ribosome, rRNA and tRNA binding), biological process (translation, secondary metabolite biosynthetic process, DNA replication and ATP synthesis coupled proton transport), cellular components (cytosol, large ribosomal subunit, and primosome complex), and pathway (ribosome, phenazine biosynthesis, quorum sensing, biofilm formation, and oxidative phosphorylation) (Figure 3a–d). While the functional enrichment analysis of DEGs for down-regulated biofilm samples revealed a single cluster (ES = 1.35) enrichment with heme biosynthetic biological process and porphyrin metabolism pathway (Figure 4).

The study conducted by DAVID yielded pathways from the Kyoto Encyclopaedia of Genes and Genomes (KEGG). The KEGG Pathways ribosome, phenazine biosynthesis, quorum sensing, and biofilm formation were shown to be significant in genes that are up-regulated in biofilm (Figure 3d).

**Figure 5:**
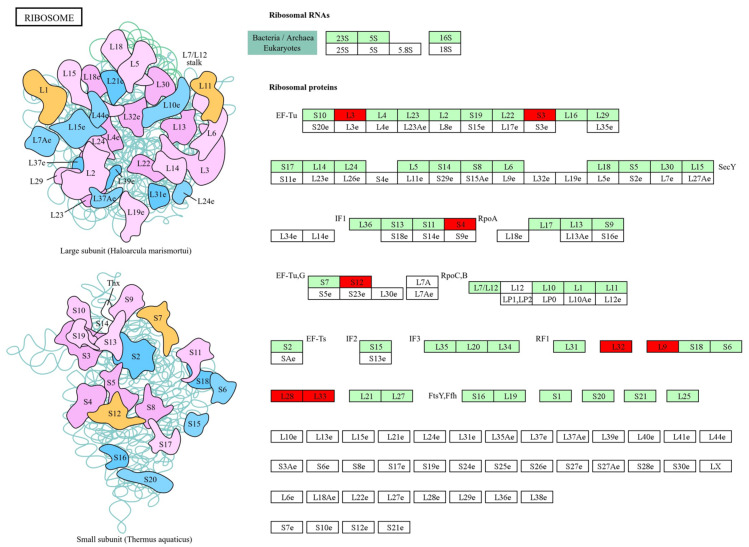
Ribosome pathway from KEGG database. Red color filed denotes genes that are up-regulated in *P. aeruginosa* biofilm formation (pae03010). Green colour filed denotes the expression of genes that are not modulated in the pathway.

**Figure 6:**
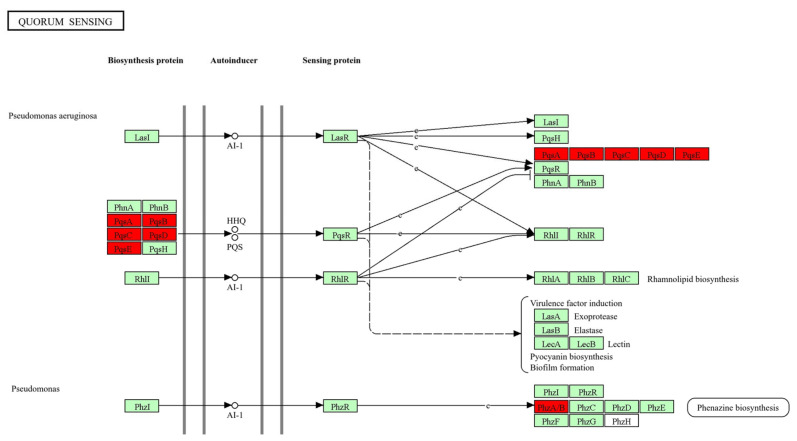
Quorum sensing pathway from KEGG database. Red color filed denotes genes that are up-regulated in *P. aeruginosa* biofilm formation (pae02024). Green colour filed denotes the expression of genes that are not modulated in the pathway.

Figure 5 displays the genes that participate in the ribosomal protein synthesis and translation that includes 5 subunits of the 50S ribosome (L3, L9, L28, L32, and L33) and 3 subunits of 30S ribosomes (S3, S4, and S12) that are up-regulated in biofilm. While the pathways phenazine biosynthesis, quorum sensing, and biofilm formation shared common genes that included PqsA, PqsB, PqsC, PqsD, PhzA, and PhzB (Figure 6).

**Figure 7:**
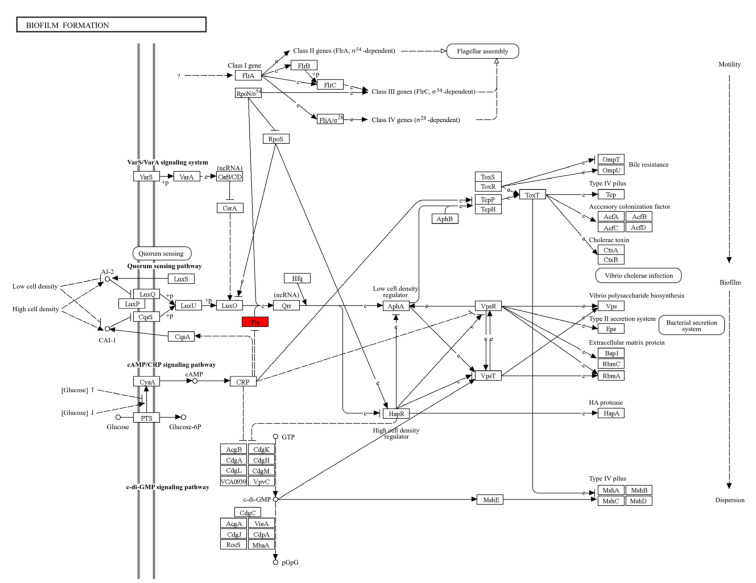
Biofilm formation pathway from KEGG database. Red color filed denotes genes that are up-regulated in *P. aeruginosa* biofilm formation (map05111).

Since the pathway associated with fis (PA4853) has not yet been reported in *P. aeruginosa*, adaptation of the biofilm pathway from Vibrio cholera revealed the up-regulated fis gene is an important contributor to biofilm formation in *P. aeruginosa* (Figure 7).

Another group of up-regulated genes in biofilm are involved in biosynthesis pyochelin (pchC, pchE, and pchG). Whereas the genes that participate in the pyoverdin biosynthetic pathway that included pvdA and pvdH were down-regulated in the biofilm of *P. aeruginosa*.

### 3.4. Structure-Based Virtual Screening

**Figure 8:**
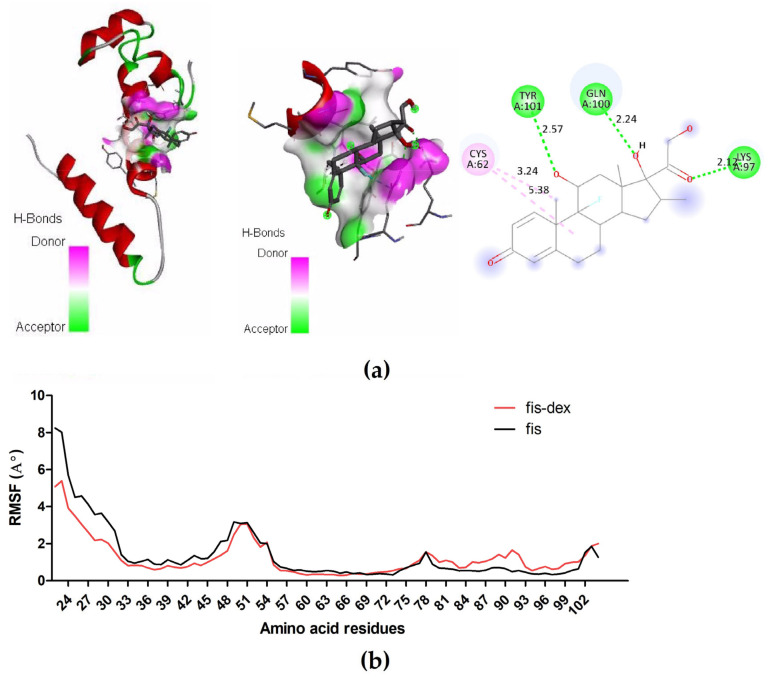
Computational analysis of dexamethasone-fis interaction. (**a**). Interaction of dexamethasone to the active site of *P. aeruginosa* factors for inversion stimulation (fis); (**b**) RMSF of the dexamethasone as well as native fis protein.

Molecular docking scores of the approved drug library show a high affinity for binding of many approved drugs toward the fis protein of *P. aeruginosa* (Supplementary Table S2). Dexamethasone was one of the best potential approved drugs that is able to prevent the enzyme from functioning at its active site (docking score—7.9 kcal/mol). Concerning the number of hydrogen bond donors/aceptors, dexamethasone is better than other drug molecules. Further analysis of the fis docked with dexamethasone revealed 3 hydrogen bonds between the receptor (Try 101, Gln 100, and Lys97) and the ligand (Figure 8). When fis was simulated using MD both alone and in combination with the optimal ligand (dexamethasone), the dexamethasone-fis complex showed a comparatively high RMSF pattern in comparison to the native enzyme, especially at residues 81–102 (Figure 8). Since there were no discernible changes in the mobility of the docked and native proteins, this may be explained by the complex’s great stability.

### 3.5. Antibacterial Activity of Dexamethasone Against P. aeruginosa

**Figure 9:**
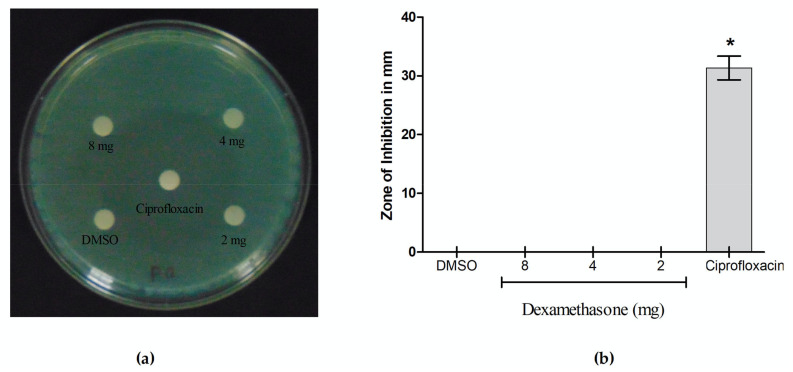
Anti-microbial potential of dexamethasone against *P. aeruginosa* investigated. (**a**) agar well disc diffusion method; (**b**) Zone of inhibition (mm) of dexamethasone and Ciprofloxacin (20 µg/well) against *P. aeruginosa*. The means ± standard error from three replicates were used to express the values, and * *p* ≤ 0.05 indicated that the results were significant.

Dexamethasone does not appear to have any antibacterial activity against *P. aeruginosa* at any of the tested doses, including the maximum concentration of 8 mg/well, according to the results of this investigation (Figure 9a,b). Furthermore, dexamethasone lacks antibacterial efficacy against *P. aeruginosa*, as demonstrated by the minimum inhibitory concentration (MIC) analysis performed with the resazurin dye reduction assay at the highest test concentration (20 mg/mL).

### 3.6. Metabolic and Biomass Inhibitory Potential of Dexamethasone Against P. aeruginosa Biofilm

**Figure 10:**
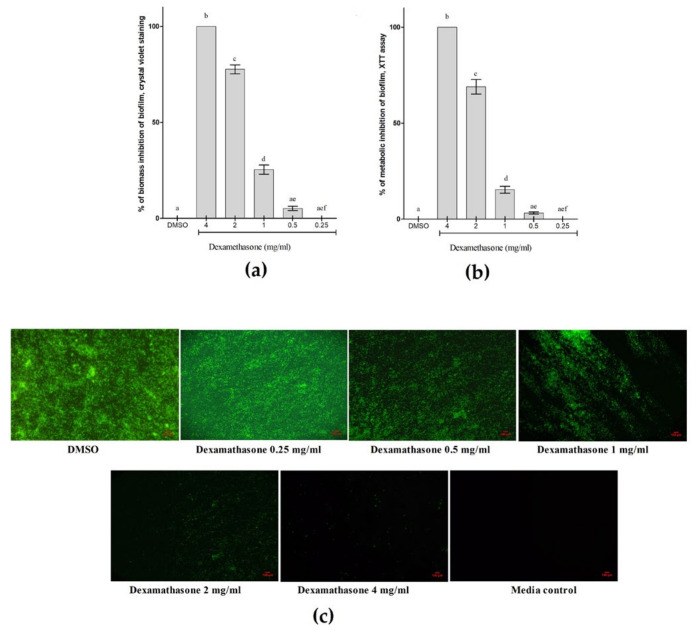
Effect of dexamethasone on *P. aeruginosa* biofilm formation. (**a**) Quantitative assessment of the *P. aeruginosa* biofilm using crystal violet staining; (**b**) Quantitative assessment of the *P. aeruginosa* biofilm using XTT reduction assay; (**c**) The *P. aeruginosa* biofilm was evaluated using acryline orange staining. The vehicle control utilized was DMSO, and the results were reported as the means ± standard error of three replicates. Findings were deemed noteworthy when * *p* < 0.05. Different superscripts b, c, d, ae, and aef, denote significant difference *p* < 0.05. (Bars labelled either a or e or f did not differ significantly).

The results showed that dexamethasone suppressed *P. aeruginosa* biofilm development in a dose-dependent pattern. By using the crystal violet staining method, it was possible to see that even at a dosage of 1 mg/mL, dexamethasone could stop *P. aeruginosa* biofilms from growing. Dexamethasone was shown to completely prevent the formation of *P. aeruginosa* biofilms at a dosage of 4 mg/mL (Figure 10a). The findings from the crystal violet staining were in agreement with the outcomes of the XTT reduction experiment. Dexamethasone-induced biofilm inhibition was shown to follow a similar pattern in the XTT reduction assay (Figure 10b). According to these results, dexamethasone may be able to prevent *P. aeruginosa* biofilms from growing in a dose-dependent way (Figure 10c). The XTT reduction experiment and the crystal violet staining method both offer proof that dexamethasone inhibits the growth of biofilms.

## 4. Discussion

The biology of *P. aeruginosa*’s ability to produce biofilms has been studied in great detail, although much remains unknown about this disease. Since biofilm structures are inherently more resistant to antibiotics, conventional treatment methods typically employed for bacterial infections are largely useless against them. Additionally complicating matters, such tactics may lead to the appearance of subpopulations that are resistant to antibiotic agents [43]. The fact that the molecular mechanism behind biofilm formation is so diverse or the inadequate methodology used in earlier research may be responsible for this knowledge gap. In addition to providing useful methods for understanding the underlying biological mechanisms, systems biology can be used to identify new treatment targets for the management and avoidance of *P. aeruginosa* biofilm formation. Transcriptomics dataset meta-analysis is an effective technique that can yield more consistent findings in selected criteria. Through the integration of expression data obtained from separate research, meta-analysis has the potential to improve a study’s statistical power and robustness [44]. The uniformity of meta-analysis study outcomes has rendered them appropriate for forecasting more dependable therapeutic targets and identifying more precise biofilm-associated pathways. Several transcriptomics investigations have been carried out thus far to distinguish the molecular mechanism connected to *P. aeruginosa* biofilm development and planktonic culture. Nevertheless, as far as we are aware, no meta-analysis investigation involving *P. aeruginosa* transcriptomics datasets has been performed on planktonic culture or biofilm forms. Integrating different sample types (treatment with antibiotics/other chemicals and gene knockout) could increase heterogeneity and have an impact on the final outcome. Hence, in order to understand the specific transcriptomic changes during biofilm formation, the gene-expression profiles associated with antibiotics/other chemical treatments and gene knockout datasets were left out in order to produce more uniform data. It is commonly discovered that biomarker studies obtained from a single experiment are less accurate due to their small sample sizes and low statistical power [24,45,46]. Therefore, to address these limitations by integrating the information and findings of several studies, a meta-analysis of related but separate investigations is carried out.

In order to compare the differences between *P. aeruginosa*’s planktonic and biofilm development forms from several public expression profiles, the current work employs meta-analysis. The variations in the organism strains and platforms that were employed were corrected using orthology mapping and random-effects modeling, respectively. A meta-analysis of differential expression revealed 83 genes using a mean effect size with an adjusted *p*-value of 0.05. The mean effect size is regarded as the differential expression calculation’s equivalent of the log2 fold change [24].

A significant number of the potential genes that were discovered were shown to serve well-established roles in the production and growth of biofilms, supporting the study’s conclusions. The top-ranked up-regulated genes are 50S ribosomal protein L28 (PA5316; rpmB), rod shape-determining protein MreC (PA4480; mreC), and translation initiation factor IF-2 (PA4744; infB) that were previously implicated in resistance to tobramycin and biofilm formation in *P. aeruginosa* [47]. In addition, similar to the present study, a gene encoding 30S ribosomal protein S3 (PA4257; rpsC) was reported to be up-regulated in biofilms of *E.coli* [48]. In addition, genes encoding ribosomal proteins rpsL, rpmG, rplI, and rplC were noticed to be up-regulated in biofilms of *P. aeruginosa*. The gene rpsL that encodes 30S ribosomal protein S12 was earlier reported to be upregulated in biofilms of *Staphylococcus aureus* [49]. Genes rpsC (50S ribosomal protein L3) and rplI (50S ribosomal protein L9) were also reported to be up-regulated in biofilms of *Haemophilus influenza* [50]. Gene expression related to ribosome activity and protein synthesis is connected with the first attachment phase and is involved in the creation of peptidoglycan, surface-associated proteins, and capsular polysaccharide/adhesion [51].

In order to defend itself during cyanogenesis, *P. aeruginosa* expresses a cyanide-insensitive terminal oxidase. Cyanide generation is toxic to nearby species. The cyanide insensitive terminal oxidase (cioB) was Overexpression of cioB prevented cyanide induced dispersal of *P. aeruginosa* biofilms [52]. It has been exposed that *P. aeruginosa* biofilms and Rhl, Las, and Pqs genes involved in QS pathways are repressed by inhibition of the uracil biosynthesis pathway [53]. The present meta-analysis of transcriptomic data revealed up-regulation of the uridylate kinase encoding gene (pyrH). An earlier investigation on *P. aeruginosa* AES-1M demonstrated increased expression of genes connected to alginate, biofilm, persistence, and virulence, including dihydroorotase, uridylate kinase, and cardiolipin synthase [54]. Expression of pyrH was reported to be up-regulated in citric acid insensitive biofilms of TctD-TctE deleted *P. aeruginosa* [55]. The present study revealed up-regulation of pchE and pchH involved in pyochelin synthesis in biofilms of *P. aeruginosa*. Recent investigations conducted in vitro reveal that DNase treatment downregulated the expression of pchE and can limit the production of biofilms by *P. aeruginosa* and *S. aureus* [56]. The pchE mutants were also unable to form biofilms and produce phenazines [57]. Peptidyl-tRNA hydrolase’s gene was up-regulated as a result of the suhB mutation. In the meantime compared to the wild-type *P. aeruginosa* strain, the suhB mutant did in fact develop biofilm at higher rates [58,59]. The above studies support the data that was reported in the present study, in which the expression of gene pth (peptidyl-tRNA hydrolase) was up-regulated in *P. aeruginosa* biofilm. Moreover, *P. aeruginosa* mutants containing transposon insertions in the tRNA pseudouridine 55 synthase (truB) gene had poor biofilms made up of tiny aggregates that had a lower biomass [60]. A nucleoid-binding protein called factor for inversion stimulation (Fis) attaches to the target gene promoter to influence gene expression widely, and its overexpression in *P. putida* revealed fis as an enhancer of biofilm formation and suppressor of dispersion of biofilm [61]. The results of this study showed that *P. aeruginosa* biofilms expressed higher levels of Fis. The Fis was reported to regulate the type III secretion system (T3SS) in *P. aeruginosa* and ciprofloxacin resistance in *P. aeruginosa* via regulation on pyocin production [62].

Type 1 fimbriae regulation and movability were critical at all stages, whereas matrix synthesis and purine biosynthesis were crucial merely as the biofilm developed. Both mobility and adherence were also critical for the early stages of the biofilm [63]. Up-regulation of the purine biosynthesis gene purH in biofilms was found to be vital and essential in the mature biofilm of *P. aeruginosa*. Disruption of de novo purine biosynthesis by mutation of purH was reported to impair biofilm production in *S. aureus* and *Enterococcus faecalis* [64]. The aspartate kinase (AK) gene lysC, which is responsible for aspartic acid phosphorylation, the initial stage of the aspartic amino-acid family’s biosynthesis, lysine, methionine, and threonine, was also found to be up-regulated in *P. aeruginosa* biofilms. Although the role of lysC with respect to biofilm formation is unknown, its mutation was predicted and reported to impair biofilm formation in *P. aeruginosa* and *Vibrio cholera* [65,66].

The sodium: solute symporter (PA3234; yjcG) that was the top-ranked down-regulated gene candidate was previously reported to be down-regulated in *P. aeruginosa* biofilm formation [47]. Kojic acid treatment up-regulated beta-alanine-pyruvate transaminase gene expression and inhibited biofilm formation in *Acinetobacter baumannii* [67]. Next to yjcG, the gene encoding Beta-alanine: pyruvate transaminase (bauA) was the most down-regulated gene in the biofilms of *P. aeruginosa*. The downregulation of the gene dnaB, which codes for a DNA helicase that can unwind long sections of double-stranded DNA, was also observed in *P. aeruginosa* biofilms. Earlier studies revealed down-regulation of dnaB in biofilms of *Streptococcus pneumonia* [68]. Similar to the present report, expression of niRN and NirH was reported to be down-regulated in biofilms of *P. aeruginosa* [69]. Throughout the biofilm-forming process, *P. aeruginosa* employs iron as a signal. The two most well-studied *P. aeruginosa* iron acquisition systems are the lower affinity pyochelin system and the high affinity pyoverdine system. Extracellular iron (Fe^3+^), which is then carried into the cell with these siderophores, is bound by pyoverdine and pyochelin [70]. The present meta-analysis of transcriptomic data revealed that genes involved in pyochelin synthesis (pchC, pchE, and pchG) were up-regulated and that genes involved in pyoverdine synthesis (pvdA and pvdH) were down-regulated in biofilms of *P. aeruginosa*. Mutation of the pvdA gene was earlier reported to abolish pyoverdine production without affecting *P. aeruginosa* biofilm formation [71]. This is in line with earlier findings by Banin et al., [68], which showed that pyoverdine by itself is not required for active iron uptake for biofilm formation [70]. Utilizing these genes will help us control and treat biofilm-based infections in addition to providing insight into further biofilm creation and development mechanisms.

Targeting bacterial DNA-binding proteins destroyed biofilms and liberated resident bacteria, promoting their eventual host immune effector clearance or antibiotics, which are now effective at significantly lower concentrations [72]. In the present study, the fis is the only DNA binding/regulatory protein that was noticed to be up-regulated in biofilms of *P. aeruginosa*. Hence, fis was used as the drug target for computational based screening of drug candidates that could inhibit biofilm formation in *P. aeruginosa*. Computational screening and docking analysis revealed that Lys 97, Try101, and Gln100 are the fis amino acids that are involved in interaction with dexamethasone. Structural analysis of *P. aeruginosa* fis protein revealed that Lys97 is an essential amino acid that is required for sequence-specific binding of fis to its target DNA site [31]. Based on the above results, it was proposed that dexamethasone could inhibit *P. aeruginosa* biofilm formation via targeting fis. In addition, the in vitro biofilm assay revealed that dexamethasone has inhibited the *P. aeruginosa* biofilm formation at sub-inhibitory concentrations. Further supporting the current findings of dexamethasone’s anti-biofilm activity towards *P. aeruginosa* biofilm is the fact that the drug previously demonstrated anti-biofilm activity against *S. aureus* [73].

## 5. Conclusions

The present study presents a robust computational way to identify key players from bacterial expression patterns by harnessing the power of meta-analysis. By comparing with other pertinent gene sets, meta-analysis has been applied for the first time in *P. aeruginosa* transcriptome investigations. To combat biofilm-forming pathogenic bacteria like *P. aeruginosa*, it is critical to comprehend the fundamental distinctions between the behaviors of planktonic and biofilm bacteria. The lack of profile samples in determining the important genes and pathways that contribute to these variations is addressed by a meta-analysis of biofilm gene expression in *P. aeruginosa*. A set of 83 candidate genes that were differentially expressed in the biofilm state were found by the investigation, along with fis as a potential therapeutic target for *P. aeruginosa* biofilm formation. These techniques were combined with meta-analysis, virtual screening, and invitro methods. Given that these genes are expected to play a significant role in biofilms, they can be used as a hallmark target list for *P. aeruginosa* anti-biofilm medications. While the study identifies potential therapeutic targets, the effectiveness and safety of these targets in a clinical setting are not validated. The translation of findings from in vitro studies and virtual screenings to clinical applications can be challenging and requires extensive testing.

## Figures and Tables

**Table 1:** RNA-Seq/microarray samples used for differential expression analysis.

S. No.	GEO Series ID	Total Number of Samples (Control: Test)	Planktonic (Control)	Biofilm (Test)	Study Platform
1	GSE30021	6 (3:3)	GSM743004, GSM743005, GSM743006	GSM743007, GSM743008, GSM743009	GPL84 [Pae_G1a] Affymetrix *Pseudomonas aeruginosa* Array
2	GSE120760	6 (3:3)	GSM3414886, GSM3414887, GSM3414888	GSM3414889, GSM3414891, GSM3414890	GPL84 [Pae_G1a] Affymetrix *Pseudomonas aeruginosa* Array
3	GSE136111	6 (3:3)	GSM4041290, GSM4041291, GSM4041292	GSM4041293, GSM4041294, GSM4041295	Illumina NextSeq 500 (*Pseudomonas aeruginosa*)
4	GSE223663	6 (3:3)	GSM6970259, GSM6970260, GSM6970261	GSM6970256, GSM6970257, GSM6970258	Illumina HiSeq 2500 (*Pseudomonas aeruginosa*)

**Table 2:** DEGs identified based on meta-analysis.

S. No.	Expression	Significance	No. of Genes
1	Down-regulated	padj 0.001	30
2	Down-regulated	padj 0.01	5
3	Down-regulated	padj 0.05	3
4	Unchanged	Unchanged	447
5	Up-regulated	padj 0.001	21
6	Up-regulated	padj 0.01	17
7	Up-regulated	padj 0.05	7

**Table 3:** List of top 25 up-regulated genes in biofilm.

Locus Tag	Gene Symbol	Gene Name	Combined Effect Size	Padj Value
PA5316	rpmB	50S ribosomal protein L28	3.01	1.57 × 10^−8^
PA4257	rpsC	30S ribosomal protein S3	2.63	4.86 × 10^−10^
PA4239	rpsD	30S ribosomal protein S4	2.47	1.58 × 10^−8^
PA3743	trmD	tRNA (guanine-N1)-methyltransferase	2.26	1.19 × 10^−8^
PA4480	mreC	rod shape-determining protein MreC	2.22	4.06 × 10^−22^
PA4933	-	hypothetical protein	2.17	1.86 × 10^−5^
PA0549	-	hypothetical protein	2.15	0.0017
PA4268	rpsL	30S ribosomal protein S12	2.12	4.32 × 10^−9^
PA3929	cioB	cyanide insensitive terminal oxidase	1.85	0.0003
PA3654	pyrH	uridylate kinase	1.81	0.0002
PA4004	-	conserved hypothetical protein	1.72	0.0028
PA4226	pchE	dihydroaeruginoic acid synthetase	1.71	0.0017
PA4223	pchH	probable ATP-binding component of ABC transporter	1.65	0.0022
PA4672	pth	peptidyl-tRNA hydrolase	1.62	5.52 × 10^−14^
PA4853	fis	DNA-binding protein Fis	1.60	0.0016
PA2970	rpmF	50S ribosomal protein L32	1.54	3.67 × 10^−11^
PA4854	purH	phosphoribosylaminoimidazolecarboxamide formyltransferase	1.54	2.52 × 10^−7^
PA5569	rnpA	ribonuclease P protein component	1.53	8.64 × 10^−10^
PA5555	atpG	ATP synthase gamma chain	1.48	0.0023
PA5315	rpmG	50S ribosomal protein L33	1.48	8.82 × 10^−12^
PA4932	rplI	50S ribosomal protein L9	1.48	1.22 × 10^−6^
PA4744	infB	translation initiation factor IF-2	1.45	0.0020
PA0904	lysC	aspartate kinase alpha and beta chain	1.42	8.08 × 10^−5^
PA4263	rplC	50S ribosomal protein L3	1.30	0.0004
PA4742	truB	tRNA pseudouridine 55 synthase	1.27	0.0006

**Table 4:** List of top 25 down-regulated genes in biofilm.

Locus Tag	Gene Symbol	Gene Name	Combined Effect Size	Padj Value
PA3234	yjcG	sodium:solute symporter	−3.04	2.34 × 10^−6^
PA0132	bauA	Beta-alanine:pyruvate transaminase	−2.95	9.56 × 10^−11^
PA4931	dnaB	replicative DNA helicase	−2.42	0.0005
PA0459	clpC	ClpA/B protease ATP binding subunit	−2.40	0.0013
PA2413	pvdH	L-2,4-diaminobutyrate:2-ketoglutarate 4-aminotransferase, PvdH	−2.22	1.40 × 10^−5^
PA3568	ymmS	acetyl-coa synthetase	−2.07	4.76 × 10^−5^
PA0509	nirN	Dihydro-Heme d1 Dehydrogenase	−2.06	2.42 × 10^−7^
PA5473	-	conserved hypothetical protein	−2.01	5.86 × 10^−8^
PA2586	gacA	response regulator GacA	−1.97	0.0001
PA5153	-	amino acid (lysine/arginine/ornithine/histidine/octopine) ABC transporter periplasmic binding protein	−1.95	0.0177
PA3091	-	hypothetical protein	−1.86	2.54 × 10^−5^
PA0747	pauC	aldehyde dehydrogenase	−1.84	1.91 × 10^−10^
PA0301	spuE	polyamine transport protein	−1.84	0.00014
PA5139	-	hypothetical protein	−1.81	0.0070
PA1716	pscC	Type III secretion outer membrane protein PscC precursor	−1.81	0.0001
PA1296	-	2-hydroxyacid dehydrogenase	−1.75	0.0006
PA2146	-	conserved hypothetical protein	−1.74	0.0007
PA0044	exoT	exoenzyme T	−1.74	0.0001
PA4571	-	cytochrome c	−1.73	1.81 × 10^−5^
PA2938	-	transporter	−1.73	4.82 × 10^−6^
PA5170	arcD	arginine/ornithine antiporter	−1.71	0.0104
PA2386	pvdA	L-ornithine N5-oxygenase	−1.71	1.45 × 10^−6^
PA3535	eprS	serine protease	−1.70	6.74 × 10^−12^
PA0512	nirH	siroheme decarboxylase subunit	−1.66	2.30 × 10^−7^
PA1940	-	hypothetical protein	−1.61	4.33 × 10^−9^

## Data Availability

Data will be made available on request.

## Supplementary Materials

The following supporting information can be downloaded at: https://www.mdpi.com/article/10.3390/cimb46110770/s1, Table S1: Meta-analysis DE; Table S2: Virtual screening.
